# Efficacy and safety of intraoperative cone-beam CT-guided localization of small pulmonary nodules

**DOI:** 10.1093/icvts/ivac236

**Published:** 2022-09-14

**Authors:** Taisuke Kaiho, Hidemi Suzuki, Atsushi Hata, Takamasa Ito, Kazuhisa Tanaka, Yuichi Sakairi, Hideyuki Kato, Yuki Shiko, Yohei Kawasaki, Ichiro Yoshino

**Affiliations:** Department of General Thoracic Surgery, Chiba University Graduate School of Medicine, Chiba, Japan; Department of General Thoracic Surgery, Chiba University Graduate School of Medicine, Chiba, Japan; Department of General Thoracic Surgery, Chiba University Graduate School of Medicine, Chiba, Japan; Department of General Thoracic Surgery, Chiba University Graduate School of Medicine, Chiba, Japan; Department of General Thoracic Surgery, Chiba University Graduate School of Medicine, Chiba, Japan; Department of General Thoracic Surgery, Chiba University Graduate School of Medicine, Chiba, Japan; Department of Radiology, Chiba University Hospital, Chiba, Japan; Biostatistics Section, Clinical Research Center, Chiba University Hospital, Chiba, Japan; Center for Preventive Medicine Sciences, Chiba University, Chiba, Japan; Department of General Thoracic Surgery, Chiba University Graduate School of Medicine, Chiba, Japan

**Keywords:** Imaging, Cone-beam computed tomography, Lung cancer surgery, Minimally invasive surgery, Thoracoscopy/video-assisted thoracoscopic surgery

## Abstract

**OBJECTIVES:**

This study aimed to evaluate the efficacy and safety of intraoperative cone-beam computed tomography-guided video-assisted thoracoscopic surgery wedge resection of impalpable small pulmonary nodules.

**METHODS:**

This was a single-centre phase 2 trial conducted between April 2018 and March 2019. Peripheral small pulmonary nodules, defined as either ground-glass opacity-dominant (>50%) nodules measuring ≤3 cm in diameter (ground-glass opacity-dominant type) or nodules measuring ≤2 cm in diameter located deeper than the nodule diameter from the visceral pleura (deep solid type), were eligible for resection using a cone-beam computed tomography-guided thoracoscopic manner. The primary end-point was macroscopic complete resection, and secondary end-points were: nodule extraction rate, operation time, localization time, marking accuracy, microscopic complete resection and safety.

**RESULTS:**

Twenty-two nodules, in 9 men and 11 women with a mean age of 64.3 years, were visualized and resected. The nodules were located in the right upper, middle and lower lobes in 3, 1 and 5 patients, respectively, and in the left upper and lower lobes in 5 and 8 patients, respectively. Seven nodules were ground-glass opacity-dominant types, and 15 were deep solid types. Cone-beam computed tomography could clearly image all nodules. The mean time for localization was 17.4 min. The mean operation time was 110.7 min. Macroscopic complete resection was accomplished in 21 nodules (95.5%). Microscopic complete resection was achieved in all nodules (100%). Postoperative air leakage and bleeding were observed in 1 patient (5%).

**CONCLUSIONS:**

Cone-beam computed tomography might be a safe and useful guide for video-assisted thoracoscopic surgery wedge resection of impalpable peripheral pulmonary nodules.

**Date and number of IRB approval:**

15 November 2017, 381.

**Clinical trial registration number:**

UMIN 000030388.

**Figure:**
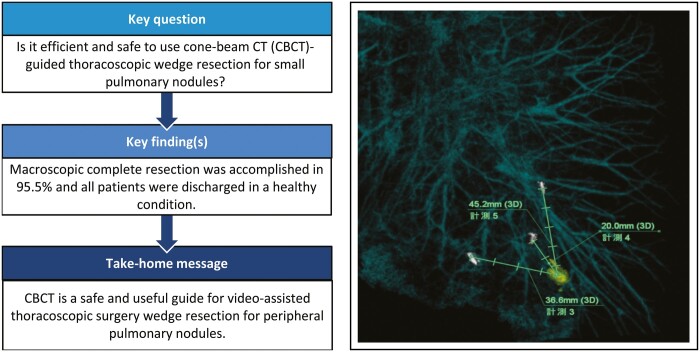

## INTRODUCTION

Advances in thoracic thin-section computed tomography (CT) and low-dose CT for detection of lung cancer are increasing the detection rate of small pulmonary nodules [1]. Two large-scale randomized phase 3 studies assessing the efficacy of low-dose CT screening in high-risk patients showed that this method of screening reduced lung cancer-related mortality [1, 2]. A large-scale Japanese study (trial JCOG0804/WJOG4507L) revealed that wide-wedge resection cures peripheral early lung adenocarcinoma, which was diagnosed radiologically as non-invasive with a consolidation/tumour ratio of ≤0.25 [3], with 5-year relapse-free survival of 99.7% and no local relapse [4]. Therefore, wedge resection could be a good option for the treatment of peripheral ground-glass opacity (GGO)-dominant lung cancer. However, such tumours are often thoracoscopically invisible and impalpable via the instruments used in video-assisted thoracoscopic surgery (VATS) and are barely palpable even in thoracotomy. Inadequate localization leads to, first, inadequate tumour margin that increases local recurrence, second, prolonged operative time due to time spent searching for nodules, and third, conversion to unplanned open thoracotomy for palpation.

To overcome this problem, various localization procedures have been reported, such as percutaneous CT-guided injection of dye [5]; wire [6] or contrast medium [7]; bronchoscopy-guided injection [8]; and electromagnetic navigation bronchoscopy-guided approaches [9]. In addition, these reported procedures were evaluated in terms of accuracy of localization, degree of invasiveness, morbidity, time-saving or -consuming, extent of radiation exposure, simple or complex and success rate of pulmonary resection of target lesions. However, to date, there have been few studies based on scientific evidence. Moreover, most of these procedures can be traumatic and/or time-consuming; invasive; and preoperative. Recently, Sato *et al.* [10] have developed a virtual-assisted lung mapping procedure: a preoperative bronchoscopic dye-marking technique under the guidance of 3-dimensional CT, in which dye is injected around the nodule region. Although the primary end-point has been set at an accurate resection rate of 95% or more, this procedure has a detection rate of 93.6% and an accurate resection rate of 87.8%. However, this procedure is complex: requires a high level of technical skill, and still does not resolve the previously mentioned problem. In addition, it essentially imposes a burden on patients as a traumatic intervention and preoperative procedure (from several hours to 2 days before surgery). Localization of pulmonary tumours using intraoperative CT has also been demonstrated by several studies [11] that aimed to overcome the long procedure time of those traumatic preoperative localization methods. CT-guided placement of wire, nylon or other materials also demonstrates highly accurate localization rates of more than 90% [12, 13]. However, placed materials sometimes migrate before the time of surgery. Moreover, these procedures are essentially traumatic and in many cases, air embolisms have been reported [14].

Identifying these gaps in the literature, we conducted a phase 2 study aimed to evaluate the utility and safety of intraoperative cone-beam CT (CBCT)-guided VATS for wedge resection of peripheral small-sized lung tumours.

## PATIENTS AND METHODS

### Ethics statement

This study was approved by the Ethics Committee at Chiba University Hospital (No. 381, issued 15 November 2017). All patients provided written informed consent prior to their participation in the study and for publication of individual patient data.

### Data availability statement

The datasets generated and analysed during the current study are available from the corresponding author on reasonable request.

#### Study plan and patients

This phase 2 clinical trial was conducted at a single institution, following the CONSORT guidelines. The patient eligibility criteria were: aged between 20 and 85 years, Eastern Cooperative Oncology Group (ECOG) performance status of 0–1, and no definitive organ dysfunction. The exclusion criteria were: pacemaker implantation; cognitive impairment; and pregnancy or breastfeeding. The eligibility criteria of nodules were: GGO-dominant (>50%) nodules with a diameter of 3 cm or less (termed GGO-dominant type); and nodules with a diameter of 2 cm or less and located deeper than the nodule diameter from the visceral pleura (termed deep solid type). Lung resection for both therapeutic and diagnostic purposes was accepted.

### Procedure

**Figure 1:**
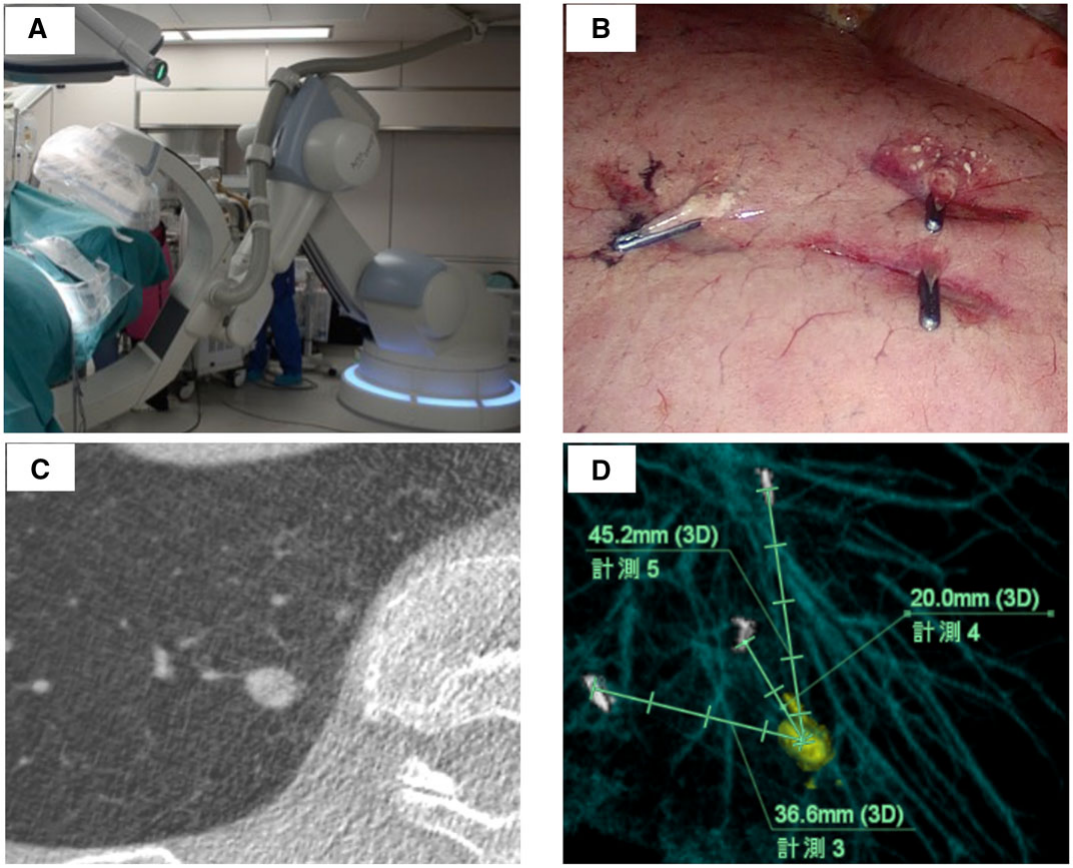
Procedure for localization of pulmonary tumor by cone-beam CT. (**A**) In a hybrid operating room, we used the cone-beam CT system Artis zeego® (Siemens, Bayern, Germany) and analyzed tumor location using a Ziostation 2® (Ziosoft, Tokyo, Japan). (**B**) Four surgical clips are attached to the visceral pleura around the suspected location of the referred tumor. (**C**) CBCT CT image of a deep solid-type tumor. (**D**) Analysis of the location of the referred tumor. CBCT: cone-beam computed tomography; CT: computed tomography.

**Figure 2:**
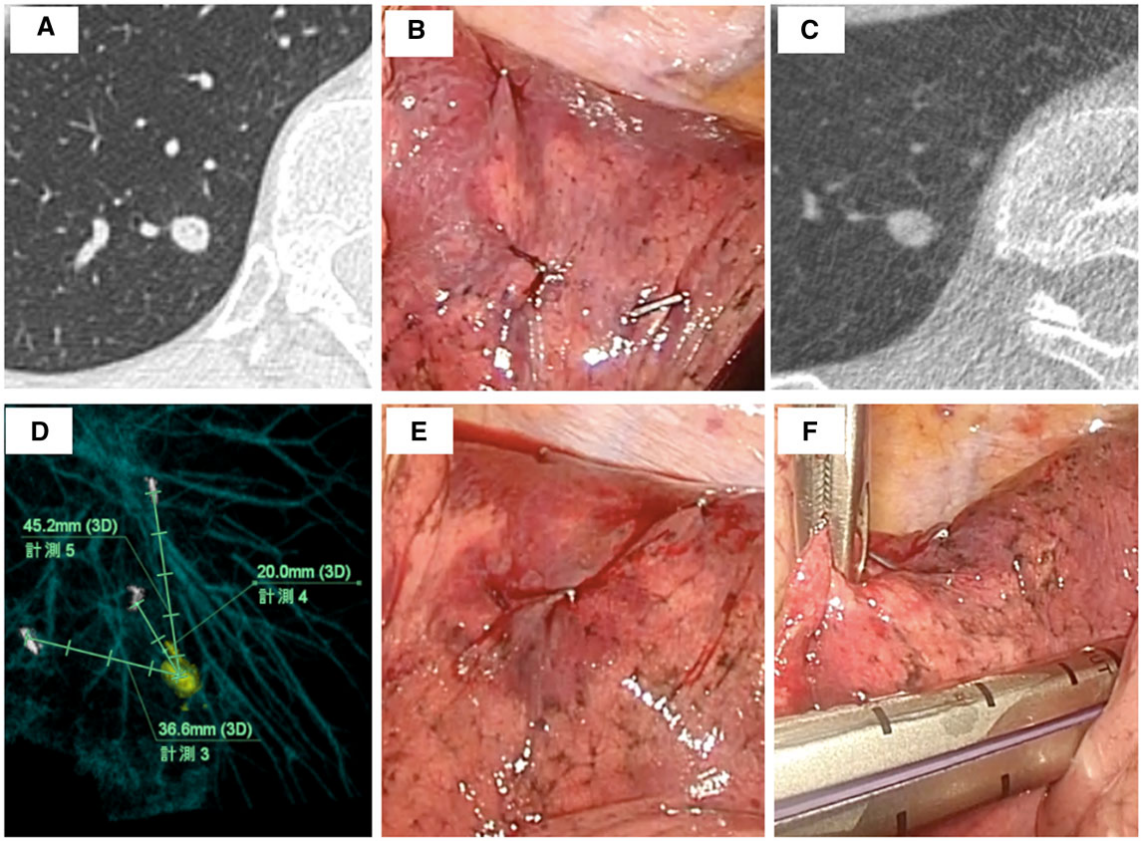
Procedure for localization of pulmonary tumour by cone-beam computed tomography. (**A**) Computed tomography image of nodule location. (**B**) Three surgical clips for first markers are attached to the visceral pleura around the suspected location of the referred tumour. (**C**) Cone-beam computed tomography image of a deep solid-type tumour. (**D**) Analysis of the location of the referred tumour. (**E**) One surgical clip for a second marker is attached to the visceral pleura above the referred tumour. (**F**) Thoracoscopic image showing wedge resection.

The protocol procedure was as follows (Fig. 1). In a hybrid operating room (OR), we used a CBCT system Artis zeego^®^ (Siemens, Bayern, Germany) and analysed tumour location using Ziostation 2^®^ (Ziosoft, Tokyo, Japan). CBCT scanning essentially utilizes a fluoroscopy C-arm that rotates around the patient and provides a real-time CT image. CBCT image quality is sufficient for localizing pulmonary nodules. An extension tube was attached to the intubation tube to create more space for anaesthesia apparatus. The CBCT arm was inserted from the left side of the patient and it was confirmed that the arm rotated in the target region before the start of operation. First, several surgical clips (2–4 clips as first markers) were attached to the visceral pleura around the nodule’s suspected location based on the preoperative CT image, using a 3-port VATS approach. The location information between the target nodule and first markers was obtained using CBCT. One surgical clip as the second marker was then attached only to the nodule location, based on images acquired with CBCT. According to this second marker and depth information from the visceral pleura obtained using CBCT, pulmonary resection was performed, guided by staplers (Fig. 2).

### End-points

The primary end-point was macroscopic complete resection (MCR) of more than 95%, defined according to the length of the resection margin as follows: 10 mm in GGO-dominant type nodules, either nodule diameter or 20 mm in deep solid-type nodules, and 5 mm for curative and diagnostic resection by VATS. The macroscopic resection margin was evaluated as the shortest distance from the tumour to the surgical margin including the length for the staple line. The secondary end-points were extraction rate of nodules, operation time, time spent on localization (i.e. time between the placement of first and second markers), distance between first markers and the target nodule, distance between second marker and target nodule, microscopic complete resection as defined by pathologists, morbidity during the operation, and by 1 month after surgery.

### Statistical analyses

We assumed an expected MCR rate of 0.95. A sample size of 37 was found to achieve a power of more than 0.80 to detect a difference of 0.15 mm using an exact 1-sided test under a null hypothesis of 0.80. Considering a dropout, we aimed to include 45 patients. Data are expressed as numbers of cases for categorical variables and means with standard deviation or median with range for continuous variables. The primary purpose of this study was to evaluate the MCR rate. The chi-squared test with a 1-sided significance level of 5% (under the null hypothesis: MCR rate is 0.80) was used to test the hypothesis. The number and rate of MCR with 90% confidence interval (CI) were calculated. There was no multiplicity adjustment for analysis of the secondary outcomes. Regarding secondary outcomes, a 1-sided significance level at 5% and the 90% CI were calculated. The hypothesis test and confidence interval calculation method were the same as those used in the primary analysis. All statistical analyses were performed using SAS 9.4 (SAS Institute Inc., Cary, NC; http://www.sas.com/en_us/legal/editorial-guidelines.html). A *P*-value < 0.05 was considered to be statistically significant.

## RESULTS

Overall, 9 men (45.0%) and 11 women (55.0%) (mean age: 64.3 years) were enrolled in this study (Table 1). The ECOG performance status was 0 in all patients; 7 were smokers and 1 had chronic obstructive pulmonary disease. Two patients had 2 nodules each. Thus, the number of evaluable nodules was 22. The mean size of the 22 nodules was 8.3 ± 3.1 mm, and the mean ratio of GGO/total size was 0.69 ± 0.44 mm. The mean distance between nodules and the visceral pleura was 8.6 ± 4.1 mm. Seven nodules (31.8%) were the GGO-dominant type, and 15 (68.2%) were the deep solid type (Table 2).

**Table 1:** Profile of patients

Parameters	Results	*n* = 20 (%)
Age (year)		
Mean [range]	64.3	[33–83]
Sex		
Male	9	(45.0)
Female	11	(55.0)
ECOG PS		
0	20	(100)
1<	0	(0)
Smoking history		
Yes	7	(35.0)
No	13	(65.0)
COPD		
Yes	1	(5.0)
No	19	(95.0)
Number of nodules		
1	18	(90.0)
2<	2	(10.0)

COPD:Chronic Obstructive Pulmonary Disease; ECOG: Eastern Cooperative Oncology Group; PS: Performance status.

**Table 2:** Characterization of pulmonary nodules

Evaluation item	Results	
Location site		
Right upper/middle/lower	3/1/5	(13.6/4.5/22.7)
Left upper/lower	5/8	(22.7/36.5)
Tumour type		
GGO dominant type	7	(31.8)
Deep solid type	15	(68.2)
Tumour size on CT (mm)		
Mean [range]	8.3 ± 3.1	[7–17]
Consolidation/tumour ratio		
Mean [range]	0.69 ± 0.44	[0–1]
Distance to the pleural space(mm)		
Mean [Range]	8.6 ± 4.1	[2–17]

CT: computed tomography; GGO: ground-glass opacity.

Results of the protocol procedure are summarized in Table 3. The mean localization time was 17.4 min (90% CI 14.5–20.2), and the mean operation time was 110.7 min (90% CI 96.4–125.0). CBCT scanning was performed twice in 11 patients to confirm location of the nodule, and the mean number of CBCT scans was 1.6 ± 0.6. All nodules and markers (100%) were clearly detected by CBCT. The mean radiation exposure dose was 68.9 ± 48.7 mGy. MCR was accomplished in 21 out of the evaluable 22 nodules (95.5%, *P *=* *0.0003, 90% CI 88.2–100), and the mean macroscopic distance of the surgical margins was 12.1 ± 3.2 mm. MCR was not achieved in 1 case with a deep solid-type nodule, diagnosed as a pulmonary metastasis from epithelioid sarcoma. The nodule size was 12 mm, and the distance between the nodule and the visceral pleura was 12 mm. The procedure was performed according to protocol, but the macroscopic distance of the surgical margin was 9 mm due to the relatively deep location of the nodule. An additional resection was not performed because of a bronchial and vascular injury risk. Additional one more wedge resection was required in 1 case with a deep solid-type nodule due to an inadequate resection margin. This case accomplished MCR. In all nodules, including the one for which MCR was not achieved, microscopic complete resection was confirmed by surgical pathology (100%, 90% CI 89.0–100), and the mean microscopic distance of the surgical margins was 9.1 ± 3.0 mm. Histologically, 11 primary lung cancer [10 adenocarcinoma (2 were in situ lesions), and 1 squamous cell carcinoma], 8 metastatic tumours (from 5 colon cancer, 1 hepatocellular carcinoma, 1 ovarian cancer and 1 epithelioid sarcoma) and 3 other types of nodules (2 solitary fibrous tumours and 1 inflammatory tissue) were identified. The mean distance between the first markers and the target nodule on the CBCT image was 21.6 ± 10.1 mm, and the mean distance between the second marker and the tumour on the resected specimen was 10.7 ± 6.6 mm. Postoperative air leakage and bleeding were observed in one each (5.0%). Postoperative air leakage occurred in one case and re-drainage was performed on postoperative day 3. Another case experienced postoperative bleeding and reoperation was performed on postoperative day 1. Bleeding was identified during wedge resection of the lung, not related to marking. This case required red blood cell transfusions. All patients were discharged from the hospital to home in a healthy condition. Open thoracotomy was required in one case for complete resection without injury to the bronchus and vessels.

**Table 3:** Results of protocol procedure

Evaluation item	Results		
Operation time (min)			
Mean (90% CI)	110.7	(96.4–125.0)	
Bleeding (ml)			
Median [range]	0		[0–85]
Localization time (min)			
Mean (90% CI)	17.4	(14.5–20.2)	
Macroscopic complete resection rate (%)			
Rate (90% CI)	95.5 (21/22)	(82.0–99.0)	
Extracting by cone-beam CT			
Yes	22/22		(100)
Distance between first marker to a tumour (A) (mm)			
Mean	21.6 ± 10.1		
Distance between second marker to a tumour (B) (mm)			
Mean	10.7 ± 6.6		
Difference between (A) and (B)(mm)			
Mean (90% CI)	10.9	(7.8–14.0)	
Numbers of cone-beam CT scanning			
Mean	1.6 ± 0.6		
Exposure dose (mGy)			
Mean	68.9 ± 48.7		
Histological types			
Primary lung adenocarcinoma (%)	10		(45.5)
Primary lung squamous cell carcinoma (%)	1		(4.5)
Metastatic carcinoma (%)	8		(36.4)
Others (%)	3		(13.6)
Macroscopic distance of the surgical margins (mm)			
Mean	12.1 ± 3.2		
Microscopic distance of the surgical margins (mm)			
Mean	9.1 ± 3.0		
Microscopic complete resection rate (%)			
Rate (90% CI)	100 (22/22)	(89.0–100)	

CI: confidence interval; CT: computed tomography.

## DISCUSSION

Localization using CBCT in a hybrid operation room is an ideal solution if this procedure enables efficient imaging and successful resection of nodules in minimal time and with minimal radiation exposure. In this study, all nodules were successfully detected by CBCT (100%). The time between setting the first and second markers was only 17 min, and the second marker was accurately positioned on all nodules. The average distance between the nodules and the second marker was only 10.7 mm, which means that the marker was almost onto the nodules because the mean depth from the visceral pleura was 8.6 mm. This accuracy is the reason for the high MCR rate of 95.5%. Even in the single case when MCR failed, the second marker was correctly set. However, in this case, the nodule was located in the deepest region (12 mm from the visceral pleura). Our method is less invasive because it does not need direct puncture of the lung parenchyma like dye injection, which reduces the risk of air embolism. In addition, the procedure is completed during surgery in a hybrid OR without preoperative and complex preparation. On the other hand, although the depth from the marking to the tumour acquired with CBCT, it could be challenging to assess the depth of the nodules intraoperatively.

In our study, the parameters are similar or superior to those of other studies, and the primary end-point of MCR was met. In VATS surgery, localization is less traumatic and quicker, with an operation time of 110.7 min and an imaging time of 17.4 min. In a hybrid OR, medical staff, including surgeons, are not exposed to radiation since the CBCT system (Artis zeego) is automated by a systemic program with a highly reliable collision protection protocol. Exposure of patients to radiation was 68.9 mGy, which is roughly equivalent to 1–2 times of that of conventional CT. In this study, the CBCT scan protocol was planned once the first marker had been placed. However, in the first 11 cases, the CBCT scan was performed twice to confirm the position of the second marker. As mentioned previously, the second marker placement was highly accurate, and a repeated scan was unnecessary and, consequently, not performed in subsequent cases. Nevertheless, radiation exposure was the only invasive component of this approach, and it is of a similar degree to procedures using CT.

Real-time localization using ultrasound might be another solution if extraction of the pulmonary lesion is possible. Although their study included palpable lesions, Kondo *et al.* [15] reported a successful intraoperative detection of GGO-harbouring lung nodules utilizing intraoperative linear type ultrasound (7.5 Hz). Recently, Ujiie *et al.* [16] have reported a preclinical analysis of various ranges of ultrasound (5.0–12.0 Hz), using clinically resected lungs for lung cancer, and found an optimal condition (10 Hz) and target (solid tumour). In their study, partly solid tumours were not easily extracted in a semi-deflated lung.

### Limitations

One limitation to our clinical study is the small number of cases. Before this phase 2 trial, we conducted a feasibility study in 11 patients to trial the marking technique. Successful localization and MCR (100%) were achieved. Therefore, we considered that a single institute study with a smaller number of cases than anticipated was adequate to prove the target value of MCR (more than 95%) in this phase 2 study. Our findings show that attaining the primary end-point was statistically significant (within a 90% CI) [17]. Further clinical trials are necessary for this technique to be accepted as a practice-based application because this strategy is not permitted by social health insurance in Japan. A second limitation is that this is a single institution study. However, with access to a hybrid OR, most thoracic surgeons would be able to perform this procedure since the marking technique is straightforward and the CBCT system is very reliable. In this clinical trial, resident surgeons operated in several cases.

## CONCLUSION

This prospective study indicates that CBCT is a relatively safe and useful guide for VATS wedge resection for impalpable peripheral pulmonary nodules.

## ABBREVIATIONS

CBCT: Cone-beam computed tomography
CI: Confidence interval
CT: Computed tomography
ECOG: Eastern Cooperative Oncology Group
GGO: Ground-glass opacity
MCR: Macroscopic complete resection
OR: Operating room
VATS: Video-assisted thoracoscopic surgery
